# Progression Patterns in Foveal-Sparing Geographic Atrophy With Double-Layer Sign Due to Neovascularization or Basal Laminar Deposits

**DOI:** 10.1167/iovs.66.11.16

**Published:** 2025-08-06

**Authors:** Serena Fragiotta, Mariacristina Parravano, Riccardo Sacconi, Maria Sole Polito, Benedetta Cioffi, Federico Rissotto, Federico Beretta, Eliana Costanzo, Enrico Romano, Vittorio Capuano, Eric H. Souied, Giuseppe Querques

**Affiliations:** 1 Ophthalmology Unit, “Sapienza” University of Rome, NESMOS Department, St. Andrea Hospital, Rome, Italy; 2 IRCCS-Fondazione Bietti, Rome, Italy; 3 Departmental Faculty of Medicine, UniCamillus-Saint Camillus International University of Health Sciences, Rome, Italy; 4 School of Medicine, Vita-Salute San Raffaele University, Milan, Italy; 5 Division of head and neck, Ophthalmology Unit, IRCCS San Raffaele Scientific Institute, Milan, Italy; 6 Ophthalmology, Centre Hospitalier Intercommunal De Creteil, Creteil, France; 7 Department of Sense Organs, Sapienza University of Rome, Rome, Italy

**Keywords:** age-related macular degeneration (AMD), double layer sign (DLS), optical coherence tomography (OCT), geographic atrophy (GA)

## Abstract

**Purpose:**

The purpose of this study was to analyze the prognostic significance of double-layer sign (DLS) in eyes with foveal-sparing geographic atrophy (GA) secondary to age-related macular degeneration.

**Methods:**

This retrospective, observational cohort study analyzed 46 eyes (46 patients) with foveal sparing GA and associated DLS, using fundus autofluorescence (FAF), near-infrared reflectance (NIR), optical coherence tomography (OCT), and OCT angiography (OCTA). DLS was defined based on OCTA findings as either thick basal laminar deposits (BLamD) or non-exudative macular neovascularization (NE-MNV). The area of GA and foveal sparing were estimated on both FAF and NIR at different time points. Centrifugal and centripetal GA growth rates referring to the lesion expansion away from and toward the fovea, respectively, were evaluated using a mathematical formula.

**Results:**

Of the 46 eyes enrolled, 25 had thick BLamD, whereas 21 had type 1 NE-MNV. The NE-MNV eyes showed significantly thicker DLS than those with BLamD (90.4 ± 39.8 µm vs. 57.0 ± 27 µm, 95% confidence interval [CI] = 0.34 to 0.78, *P* < 0.001). GA areas were smaller on FAF than NIR (95% CI = −0.89 to −0.03, *P* = 0.03) in the BLamD group, whereas no difference was observed in the NE-MNV group (95% CI = −0.37 to 0.64, *P* = 0.60). Despite similar GA areas, the NE-MNV eyes exhibited larger foveal sparing (95% CI = 0.02 to 1.21, *P* = 0.04). The foveal sparing area remained stable (F(1.2, 11 = 4.15, *P* = 0.06, ω^2^ = 0.02) in the NE-MNV group, whereas a significant reduction was observed in the BLamD subgroup (F(1.39, 20.9) = 7.5, *P* < 0.001, ω^2^ = 0.09).

**Conclusions:**

OCTA has provided valuable insights into the pathogenic interpretation of the DLS signature. Our findings confirm that a neovascular DLS protects the retinal pigment epithelium and outer retina, contributing to prolonged foveal preservation.

Geographic atrophy (GA) is a late-stage complication of age-related macular degeneration (AMD), with prevalence expected to rise significantly in the coming decades.^1^ Pathologic structural alterations in GA included loss of photoreceptors, retinal pigment epithelium (RPE), and choriocapillaris, leading to irreversible functional impairment.^2^^–^^5^ The atrophic complication significantly impairs daily activities, such as reading, driving, recognizing faces, and household tasks, while also affecting financial and social well-being. Notably, visual acuity is an unreliable indicator of the disease’s functional impact.^6^^,^^7^ In fact, in about half of the cases, atrophy can develop in a horseshoe pattern initially sparing the fovea with a relative preservation of visual acuity. As the disease progresses, atrophic changes involve the fovea with a variable progression rate.^8^^,^^9^ Therefore, the preservation of foveal sparing represents a critical prognostic factor, sparking scientific debate on potential protective signatures. Notably, the role of double layer sign (DLS) in foveal sparing has gained attention as an influential factor in the GA growth rate.^10^ This signature is characterized by a distinct separation between the RPE and its basal lamina from Bruch's membrane (BrM), which becomes visible as a thin hyper-reflective line parallel to the hyper-reflective RPE.^11^^,^^12^ The protective effect of DLS has been attributed to the presence of non-exudative macular neovascularization (NE-MNV), which may support the ischemic choriocapillaris and hypoxic outer retina.^10^^,^^13^^–^^16^ However, recent evidence has raised discrepancies, attributing to thin DLS, a predictive role toward the progression of complete RPE and outer retina atrophy (cRORA) growth.^17^^,^^18^ The discrepancies in the interpretation of DLS as either a protective or detrimental feature in atrophy progression stem from its ambiguous interpretation. A distinction between thin and thick DLS—characterized by the presence of at least two hyper-reflective layers between the RPE and BrM—has helped clarify this duality. Thick DLS has been identified as a strong baseline predictor of neovascular conversion, whereas thin DLS does not appear to increase the risk of developing MNV. This supports a potential pathogenic distinction, where thin DLS may correspond to thick basal laminar deposits (BLamD), which are hypothesized to be more likely associated with an increased risk of atrophy progression.^19^

Recent histopathological evidence provides crucial insights into the significance of DLS, confirming that this optical coherence tomography (OCT)-visible feature may represent either an NE-MNV or thick BLamD.^20^ Differentiating between these distinct entities can be challenging when relying solely on structural OCT.^11^ In this context, OCT angiography (OCTA) offers high sensitivity (81.8%) and specificity (100%) for the detection of NE-MNV.^21^ With ongoing advancements in OCTA technology—particularly in resolution and scanning speed—the identification of NE-MNV has become more accurate, overcoming previous technical limitations.^16^

The present study aims to analyze the progression rate of foveal sparing GA/cRORA associated with either NE-MNV or thick BLamD. To ensure an accurate distinction between these two clinical entities, their differentiation was ascertained through OCTA. Furthermore, this study provides information on the pattern of atrophy expansion and the longitudinal changes in the foveal sparing region.

## Methods

The present retrospective cohort study included patients with GA secondary to AMD with foveal sparing and at least 1 year of follow-up. Patients were enrolled between September 2022 and June 2023 at 3 tertiary care centers, including the Medical Retina and Imaging Unit, the Department of Ophthalmology, IRCCS San Raffaele Scientific Institute (Milan, Italy); the IRCCS-Bietti Foundation (Rome, Italy); and the Centre Hospitalier Intercommunal De Creteil (Creteil, France). The study was conducted according to the Declaration of Helsinki (1975) and its following amendments. The research protocol received Institutional Review Board (IRB) approval from the IRCSS-Fondazione Bietti committee. Informed consent was obtained from all the participants in the study.

Patients were included if they had complete demographic and medical records, OCT with near-infrared reflectance (NIR), fundus autofluorescence (FAF), and OCTA at baseline and at each subsequent follow-up. The diagnosis of GA was made by senior retinal specialists at each institution (authors G.Q., M.P., and V.C.) based on clinical and multimodal imaging features (FAF, NIR, and OCT), and was consistently confirmed across all modalities and follow-up visits. By including only cases with unequivocal GA features on all imaging modalities, the risk of inter-modality discrepancies or diagnostic uncertainty among readers was minimized.^22^ For structural OCT definition, CAM criteria^23^ (Classification of Atrophy Report no. 3) were applied, thus considering cRORA as a zone of attenuation or disruption of the outer retina and RPE and a region of underneath choroidal hypertransmission of at least 250 µm.

Exclusion criteria were as follows: (a) ocular media opacities interfering with imaging acquisition; (b) the presence of known drusenoid pigment epithelium detachment (dPED); (c) a medical history of intravitreal injections or vitreoretinal surgery; (d) the presence of RPE apertures, any RPE discontinuities (e.g. micro-rips, rips, or tears), or evidence of RPE rippling or retraction at any follow-up time; (e) other possible causes of atrophy not secondary to AMD (e.g. inherited retinal dystrophies, pachychoroid syndrome, pattern dystrophy, etc.); (f) the use of systemic drugs that may cause retinal toxicity (e.g. hydroxychloroquine, thioridazine, pentosan polysulfate sodium, and dideoxyinosine); and (g) poor quality images at baseline or any follow-up visit, including a signal strength index (SSI) below 8 for PLEX Elite, a signal-to-noise ratio below 25 decibels (dB) for Spectralis OCTA, or the presence of motion artifacts.

### Multimodal Imaging Features and Their Characterization

All patients underwent spectral-domain OCT through Heidelberg Spectralis OCT (HRA + OCT; Heidelberg Engineering, Heidelberg, Germany), with a standardized scanning protocol of 25 lines distributed in a pattern size of 20 degrees × 20 degrees centered on the fovea. The distance between consecutive B-scans was 258 µm. The images were acquired using the Automatic Real Time (ART) mode and the follow-ups were taken selecting the progression tool of the Spectralis built-in software. Near-infrared reflectance was obtained using a default scanning angle of 30 degrees during the OCT acquisition. The same instrument (Spectralis HRA + OCT) was used to acquire FAF images within the same session, using an acquisition pattern of 30 degrees × 30 degrees centered on the fovea. The instrument specifications reported an excitation wavelength of 488 nm and a barrier filter transmitted light from 500 to 680 nm.

OCTA was obtained with either Heidelberg Spectralis (Heidelberg, Germany) or PLEX Elite 9000 (Carl Zeiss Meditec, Inc., Dublin, CA, USA) to confirm the presence of MNV within the RPE-basal lamina (BL)-BrM splitting. OCTA was used solely for MNV confirmation, without inter-device quantitative comparisons, as prior studies have shown these systems to be comparable in their ability to detect MNV.^24^^,^^25^

**Figure 1.**
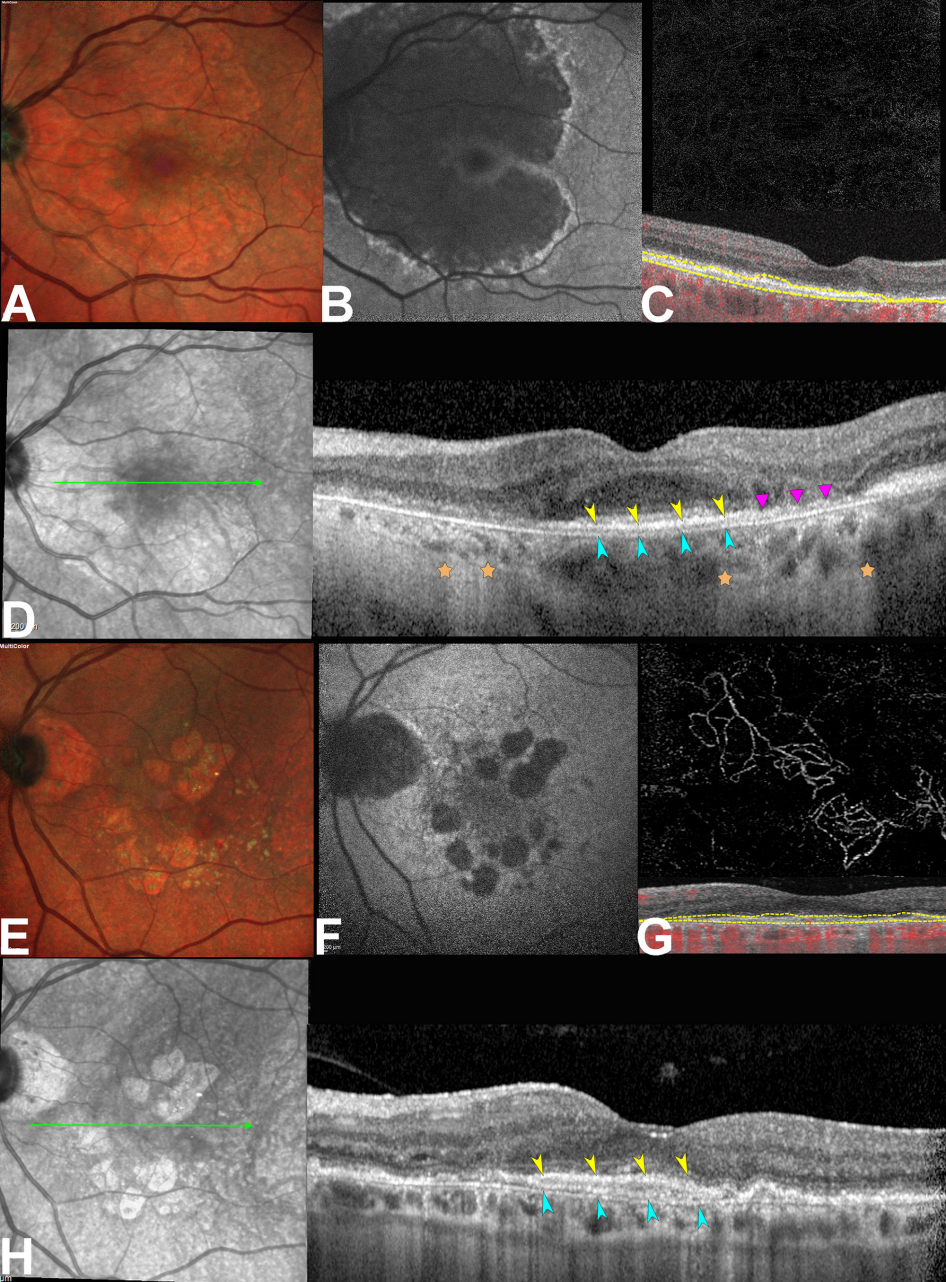
**Multimodal imaging distinction of double layer sign (DLS).** Thin DLS. (**A**) Multicolor imaging obtained through Spectralis optical coherence tomography (HRA + OCT; Heidelberg Engineering, Heidelberg, Germany). (**B**) Fundus autofluorescence (FAF) shows the extension of the hypofluorescent area corresponding to the outer retina and retinal pigment epithelium (RPE) atrophy. (**C**) OCT angiography (PLEX Elite 9000; Carl Zeiss Meditec, Inc., Dublin, CA, USA) demonstrated the absence of flow signal using an RPE-RPE fit segmentation. (**D**) OCT B-scan passing through the fovea (*green arrow*) reveals the presence of a thin RPE-basal lamina (BL; *yellow arrowheads*) Bruch's membrane (BrM; *teal arrowheads*) splitting with a hyporeflective interior. A residual hyper-reflective coarse material can be appreciated in correspondence with a nascent atrophic region (*magenta triangles*), likely expression of residual basal laminar deposits (BLamD). The posterior hypertransmission regions are marked by *orange stars*. (**E**) Multicolor imaging of a case with thick BLamD. (**F**) The corresponding demonstrated hypo-autofluorescent oval regions of atrophy surrounding the fovea. (**G)** OCT angiography demonstrated the presence of a type 1 neovascular network using an RPE-RPE fit segmentation. (**H**) OCT B-scan passing through the fovea (*green arrow*) demonstrates the presence of a thick separation between the RPE-BL (*yellow arrowheads*) and the BrM (*teal arrowheads*) with a stratified reflectivity.

The presence of DLS was defined as an RPE-BL elevation with an evident separation from the BrM. As demonstrated from a recent clinicopathologic correlation,^20^ a DLS signature can represent either thick BLamD or a type 1 MNV in a non-exudative state. To distinguish between these two pathologic entities, the presence of a thin hyporeflective DLS with a homogenous interior and the absence of a neovascular network on OCTA was considered as thick BLamD, whereas the presence of a thick multilayered DLS or triple layer sign^26^ with evidence of a neovascular flow signal on OCTA was considered as MNV.^11^^,^^17^^,^^18^ The DLS signature was assessed in the area of foveal sparing, and classified as: (a) Thick BLamD: thin DLS without evidence of flow on OCTA, or (b) NE-MNV: thick DLS with evidence of a neovascular network on OCTA (Fig. 1). An arbitrary thickness cutoff to distinguish thin from thick DLS has not yet been established. Therefore, the distinction in our study was primarily based on internal reflectivity, as demonstrated in previous studies,^17^^,^^19^^,^^20^ and was further supported by the use of OCTA.

The point of maximum vertical and horizontal diameter of DLS was estimated using the digital caliber provided by the Spectralis built-in software (software version 1.10.2.0).

All the qualitative and quantitative features were acquired by experienced retinal specialists at their respective tertiary centers (authors S.F., R.S., and V.C.). Each specialist assessed only the images from their own center. In case of disagreement or uncertainty, the qualitative features and measurements were reviewed thorugh open adjudication by senior retinal specialists (authors M.P., G.Q., and E.S.).

### Geographic Atrophy Growth and Progression Rate

Foveal sparing GA was defined as the absence of atrophic changes within the fovea, assessed using multimodal imaging. The primary modality was FAF, where the sparing was identified by the preservation of normal autofluorescence signal. NIR imaging was also used, with sparing defined by preserved reflectivity at the same topographical level. Structural OCT served as a confirmatory tool with a dual role, (i) to assess foveal sparing based on the presence of an intact ellipsoid zone and RPE layer using the foveal depression as a landmark, and (ii) to evaluate the presence and phenotypic characteristics of the DLS. The quantification of foveal sparing and GA areas was estimated through a manual delineation using both FAF and NIR at baseline and at the last available follow-up. NIR was also used as a reference to delineate the foveal sparing area to overcome the reduced FAF signal due to the absence of fluorophores in the areas with RPE atrophy.^10^^,^^27^^,^^28^ The patterns of GA growth included the (i) GA growth rate expressed as mm^2^/year, representing the yearly change or growth of the lesion area; (ii) centripetal progression (mm^2^), indicating the area of atrophy spreading toward the fovea; and (iii) centrifugal progression (mm^2^), denoting the area of atrophy spreading toward the periphery. The centripetal and centrifugal progression were calculated using the following formulas:

**Centripetal growth (mm^2^):**

$$\begin{array}{c} \frac{\begin{array}{c} \mathrm{Foveal}\;sparing\;\mathrm{area}\;\left(mm^{2}\right)at\;\mathrm{last}\;visit\\ \;-\;\mathrm{Foveal}\;\mathrm{sparing}\;area\;\left(mm^{2}\right)at\;\mathrm{first}\;\mathrm{visit} \end{array}}{Last\;visit-First\;visit\;\left(years\right)} \end{array}$$

**Centrifugal growth (mm^2^):**

$$\begin{array}{c} \frac{\begin{array}{c} \mathrm{Foveal}\;\mathrm{sparing}+GA\;area\;\left(mm^{2}\right)at\;last\;visit\\ \;-\mathrm{Foveal}\;sparing\;\mathrm{area}+GA\;area\;\left(mm^{2}\right)at\;\mathrm{first}\;visit \end{array}}{Last\;visit-\mathrm{First}\;visit\;\left(years\right)} \end{array}$$

The areas of foveal sparing and GA were converted using the square root transformation to mitigate the relationship between the baseline lesion size and the growth rates.^29^ The analysis of GA lesion growth, patterns of atrophy expansion, and foveal sparing were performed on the 1-year progression rate (T1), but when available the latest follow-up (T2) was also analyzed.

### Statistical Analysis

Quantitative data are presented as mean ± standard deviation (SD) after verifying normality using the Shapiro-Wilk test. Fisher’s exact test or the chi-square test was used to assess differences among categorical variables, as appropriate. For continuous variables, differences between groups were analyzed using unpaired *t*-tests or Mann-Whitney *U* tests (equivalent to the Wilcoxon rank-sum test). Equality of variances was evaluated with Levene’s test; if significant (*P* < 0.05), Welch’s correction was applied to adjust the *P* value. For paired data, paired *t*-tests or Wilcoxon signed-rank tests were used after verifying the normality of distribution. To identify the optimal cutoff value for DLS thickness, the diagnostic performance of various threshold values was evaluated using the cutpointr package (version 1.1.2; RStudio 2022.07.1). This statistical tool automatically computes sensitivity, specificity, and Youden's Index, defined as: Youden's Index = (sensitivity + specificity) – 1.^30^ The index ranges from 0 to 1, where 1 indicates perfect diagnostic accuracy, and reflects the overall discriminative power of each thickness cutoff. The analysis of different time points was performed using repeated measures ANOVA with Mauchly’s Test of Sphericity to assess sphericity. If the assumption was violated (*P* < 0.05), the Greenhouse-Geisser correction was applied. Omega squared (ω^2^) was used as unbiased measure of effect size in consideration of the small sample size (*n* < 30). Bland-Altmann statistic was used to test the level of agreement between the areas of GA measured on FAF and NIR.

The significance threshold for all analyses was set at *P* < 0.05 (2-sided). Statistical analyses were conducted using RStudio software, version 2022.07.1 (RStudio; PBC, Boston, MA, http://www.rstudio.com/).

## Results

A total of 46 eyes from 46 subjects presenting GA with foveal sparing fulfilled the inclusion criteria for the study. Overall, the enrolled population (40 women and 6 men) presented a mean age of 79.5 ± 11.1 years. Of these 46 eyes, 25 eyes presented a DLS without evidence of MNV on OCTA and were therefore classified as thick BLamD, whereas the remaining 21 eyes presented a DLS associated with NE-MNV. There were no disagreements between readers in the classification of BLamD versus NE-MNV, as all cases were confirmed using OCTA. The main demographic and baseline data of the populations considered are reported in Table 1.

**Table 1.** Demographic and Baseline Characteristics of the Groups at Baseline

	BLamD (*N* = 25)	NE-MNV (*N* = 21)	95% CI	*P* Value
Gender, F (%)	19 (76)	17 (80.9)	−0.32 to 3.86	0.08
Age	77.1 ± 7.3	82.5 ± 13.9	−0.09 to 1.1	0.10
LogMAR	0.27 ± 0.26	0.33 ± 0.33	−0.49 to 0.29	0.49
Snellen equivalent	20/40	20/40		

*N*, number; BLamD, basal laminar deposits; 95% CI, 95% confidence interval; LogMAR, logarithm of the minimum angle of resolution; NE-MNV, non-exudative macular neovascularization. Quantitative data are expressed as mean ± standard deviation, unless otherwise specified.

### Morphometric Characteristics of Atrophic Lesions and Double Layer Sign

Eyes with NE-MNV presented a higher RPE-BL-BrM splitting (i.e. DLS) compared to the DLS subgroup from thick BLamD at baseline (95% confidence interval [CI] = 0.34 to 0.78, *P* < 0.001). No significant differences were evident in terms of horizontal diameter between groups (95% CI = −0.29 to 0.36, *P* = 0.80) as reported in Table 2. Therefore, the DLS height was used to determine the discriminative power of arbitrary cutoff, which were chosen based on the thickness value distribution and summarized in Table 3. A cutoff of <80 µm provided the best overall balance between sensitivity (92%) and specificity (57.1%) for identifying eyes with thick BLamD, yielding the highest Youden's index of 0.49. Higher thresholds such as <100 µm and <90 µm achieved very high sensitivity (96%) but at the cost of reduced specificity (33.3% and 42.8%, respectively). Conversely, lower thresholds such as <60 µm and <50 µm improved specificity (85.7% and 90%), but sensitivity decreased to 60% and 52%, respectively.

**Table 2.** Baseline Morphometric Characteristics of the Atrophy and Double Layer Sign Between Groups

	BLamD (*N* = 25)	NE-MNV (*N* = 21)	95% CI	P Value
DLS, height, µm	57 ± 27	90.4 ± 39.8	0.34 to 0.78	<0.001
DLS, width, µm	2009 ± 889.7	2099 ± 989	−0.29 to 0.36	0.80
GA area on FAF, mm^2^	8.9 ± 7.5	10.3 ± 8.9	−0.24 to 0.41	0.60^*^
Foveal sparing on FAF, mm^2^	1.4 ± 0.71	2.1 ± 1.4	0.02 to 1.21	0.04
GA area on NIR, mm^2^	10.2 ± 7.4	9.7 ± 8.9	−0.59 to 0.35	0.61^*^
Foveal sparing on NIR, mm^2^	1.7 ± 0.8	2.8 ± 1.7	−0.21 to 0.48	0.41^*^

DLS, double layer sign; FAF, fundus autofluorescence; GA, geographic atrophy; NIR, near-infrared reflectance.

* Mann-Whitney *U* test.

**Table 3.** Diagnostic Performance of Thickness Value Cutoff to Identify Eyes With Thick Basal Laminar Deposits

Thickness Height	Sensitivity	Specificity	Youden's Index
<100 µm	96%	33.3%	0.29
<90 µm	96%	42.8%	0.39
<80 µm	92%	57.1%	0.49
<70 µm	68%	71.4	0.39
<60 µm	60%	85.7%	0.46
<50 µm	52%	90%	0.43

µm, micrometers.

Geographic atrophy areas at baseline evaluated on FAF showed no significant differences between groups (95% CI = −0.24 to 0.41, *P* = 0.60), whereas foveal sparing was greater in eyes with NE-MNV compared to thick BLamD (95% CI = 0.02 to 1.21, *P* = 0.04). Table 2 summarizes the main morphometric characteristics between the groups.

**Figure 2.**
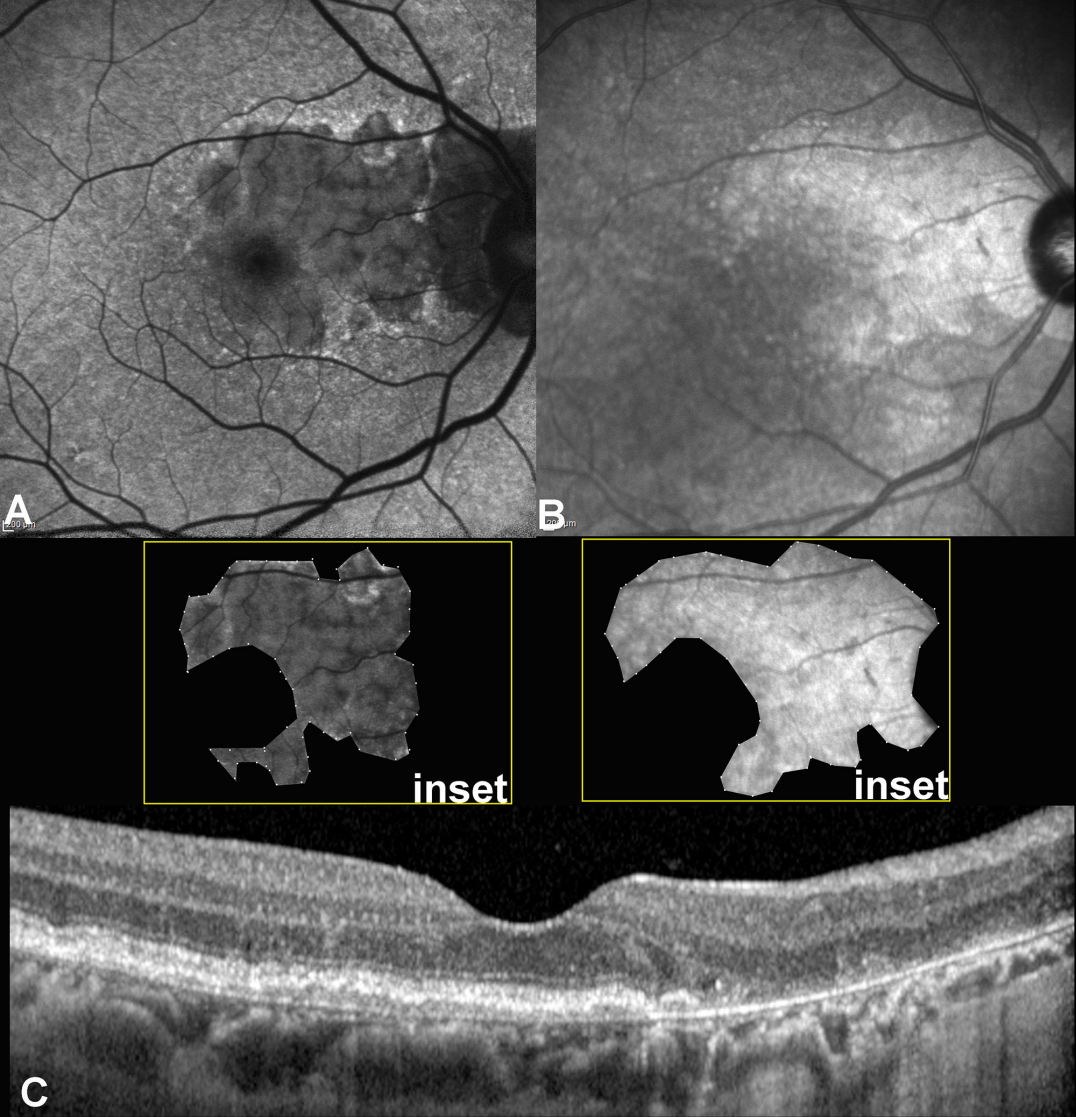
Double layer sign (DLS) associated with thick basal laminar deposits. (**A**) Fundus autofluorescence (FAF; Heidelberg Engineering, Heidelberg, Germany) shows the extension of geographic atrophy (GA) seen as a hypo-autofluorescent area surrounded by a ring of increased autofluorescence. (**B**) Near-infrared reflectance (NIR) obtained during the same visit shows the atrophic region as hyper-reflective. The atrophic area at baseline appears different from those observed on NIR, with an apparent underestimation of atrophy on FAF at baseline (*insets*). Despite this, the Bland-Altmann test revealed a substantial agreement between the two imaging methods, as shown in Figure 3. (**C**) Spectral-domain optical coherence tomography (OCT) B-scan passing through the fovea shows a thin peculiar separation of the retinal pigment epithelium (RPE) and its basal lamina with Bruch’s membrane with a hyporeflective homogenous interior.

**Figure 3.**
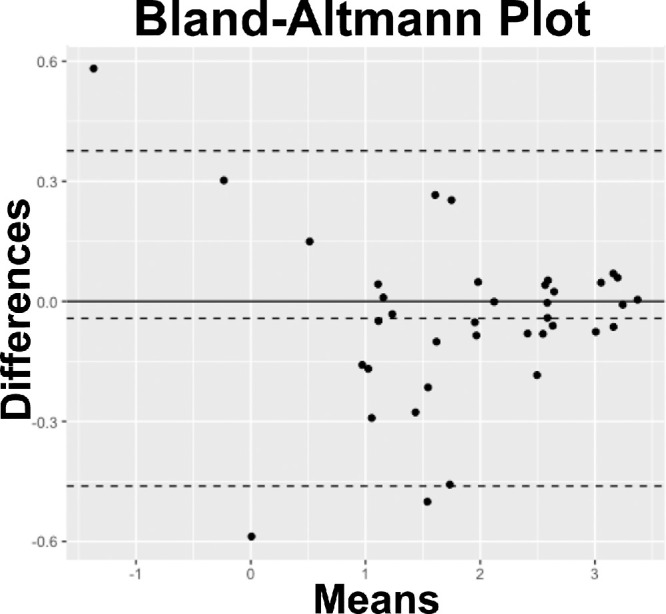
Bland Altmann plot analyzing the geographic atrophy (GA) area delineation on both fundus autofluorescence and near-infrared images. The bland-Altmann test revealed a substantial agreement between the two methods of GA areas delineation (mean bias = −0.27, 95% CI = 1.7 to −2.25, *P* = 0.10).

The GA areas in the BLamD subgroup were significantly different when measured on FAF and NIR, with an apparent underestimation when delineated on FAF (95% CI = −0.89 to −0.03, *P* = 0.03), whereas the areas of GA did not show any significant difference in the NE-MNV subgroup (95% CI = −0.37 to 0.64, *P* = 0.60). For further details, see Figure 2. No significant differences in the foveal sparing area between FAF and NIR were noted in either the BLamD group (95% CI = −0.60 to 0.30, *P* = 0.78) or the NE-MNV group (95% CI = −0.69 to 0.20, *P* = 0.51). However, the Bland-Altmann test revealed a substantial agreement between the two methods of GA area delineation (mean bias = −0.27, 95% CI = 1.7 to −2.25, *P* = 0.10) and after log-transformation (mean bias = −0.04, 95% CI =0.36 to −0.46, *P* = 0.22). Figure 3 reports the graphic plot for Bland-Altmann statistics.

### Longitudinal Changes in Geographic Atrophic Growth in DLS

After 1 year of follow-up, BCVA was stable in NE-MNV (95% CI = −0.34 to 0.80, *P* = 0.43) and with a trend toward a worsening in the BLamD subgroup not reaching the statistical significance (95% CI = −0.98 to 0.02, *P* = 0.06). Table 4 reports the average parameters after 1-year follow-up for both groups. The two groups showed no differences in terms of average GA growth rate, area of foveal sparing, and centripetal and centrifugal lesion expansions at 1 year.

**Table 4.** Longitudinal Changes in Eyes With Double Layer Sign After 1 Year

	BLamD (*N* = 25)	NE-MNV (*N* = 21)	95% CI	P Value
LogMAR at 1 y	0.33 ± 0.33	0.28 ± 0.22	−0.84 to 0.52	0.55
SQRT GA rate, mm	1.74 ± 0.60	1.58 ± 0.75	−0.81 to 0.35	0.44
SQRT centripetal, mm	0.23 ± 0.3	0.15 ± 0.23	−0.34 to 0.82	0.42
SQRT centrifugal, mm	0.46 ± 0.32	0.37 ± 0.29	−0.86 to 0.30	0.34
GA rate, mm^2^	3.4 ± 2.2	3 ± 2.8	−0.48 to 0.15	0.28^*^
Area of foveal sparing, mm^2^	1.2 ± 0.8	1.9 ± 1.4	−0.06 to 0.56	0.11^*^

NE-MNV, non-exudative macular neovascularization; SQRT, square root GA.

* Mann-Whitney *U* test (*P* < 0.05).

The total follow-up time was 2.4 ± 1.9 years for BLamD and 2.3 ± 0.86 years for NE-MNV without relevant differences between the groups (95% CI = −0.83 to 0.67, *P* = 0.83). After a mean of 36.4 ± 24.9 months, 5 of 21 (23.8%) eyes of the NE-MNV subgroup developed exudative changes, whereas none of the eyes in the BLamD subgroup progressed to exudative MNV.

**Figure 4.**
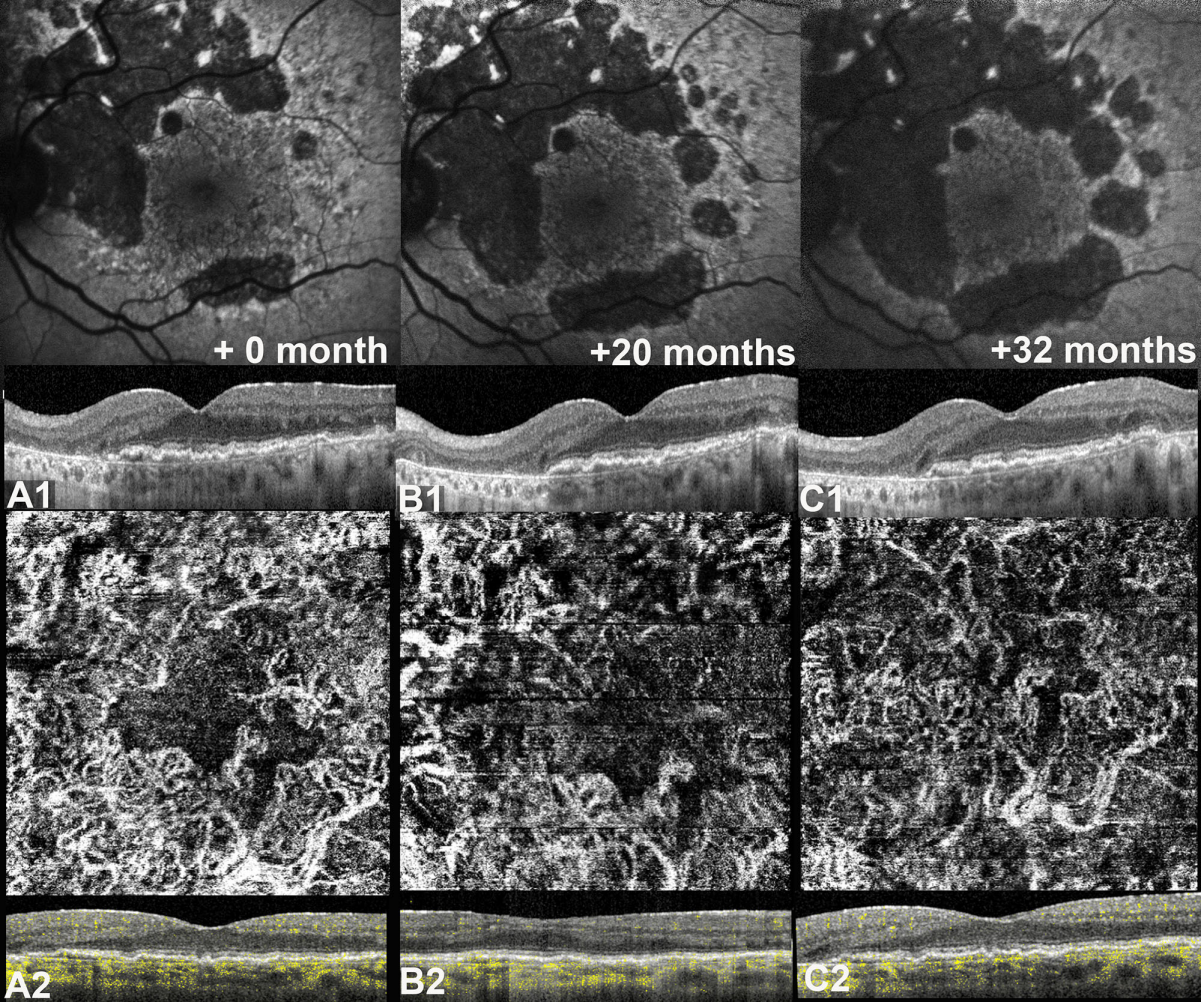
Geographic atrophy (GA) with foveal sparing in a case of double layer sign (DLS) associated with non-exudative macular neovascularization (NE-MNV). (**A1**) Fundus autofluorescence (FAF) at baseline shows the atrophic lesion with foveal sparing as confirmed through optical coherence tomography (OCT) B-scan passing through the fovea. The OCT B-scan is showing an irregular DLS at medium internal reflectivity. (**A2**) OCT angiography (Heidelberg, Germany) of the choriocapillaris slab confirms the presence of a type 1 neovascular network at baseline. (**B1**) At 20 months, the atrophic area has enlarged with relative preservation of the macular region, corresponding to the area occupied by a thick DLS on OCT B-scan passing through the fovea. (**B2**) OCTA confirmed the presence of a neovascular signal within the DLS. (**C1**) At 32 months from baseline, despite a further enlargement of the atrophic lesion can be appreciated, the foveal sparing area is still preserved and relatively stable compared to **B1**. (**C2**) OCTA slab demonstrates the presence of a diffuse neovascular network within the DLS.

**Figure 5.**
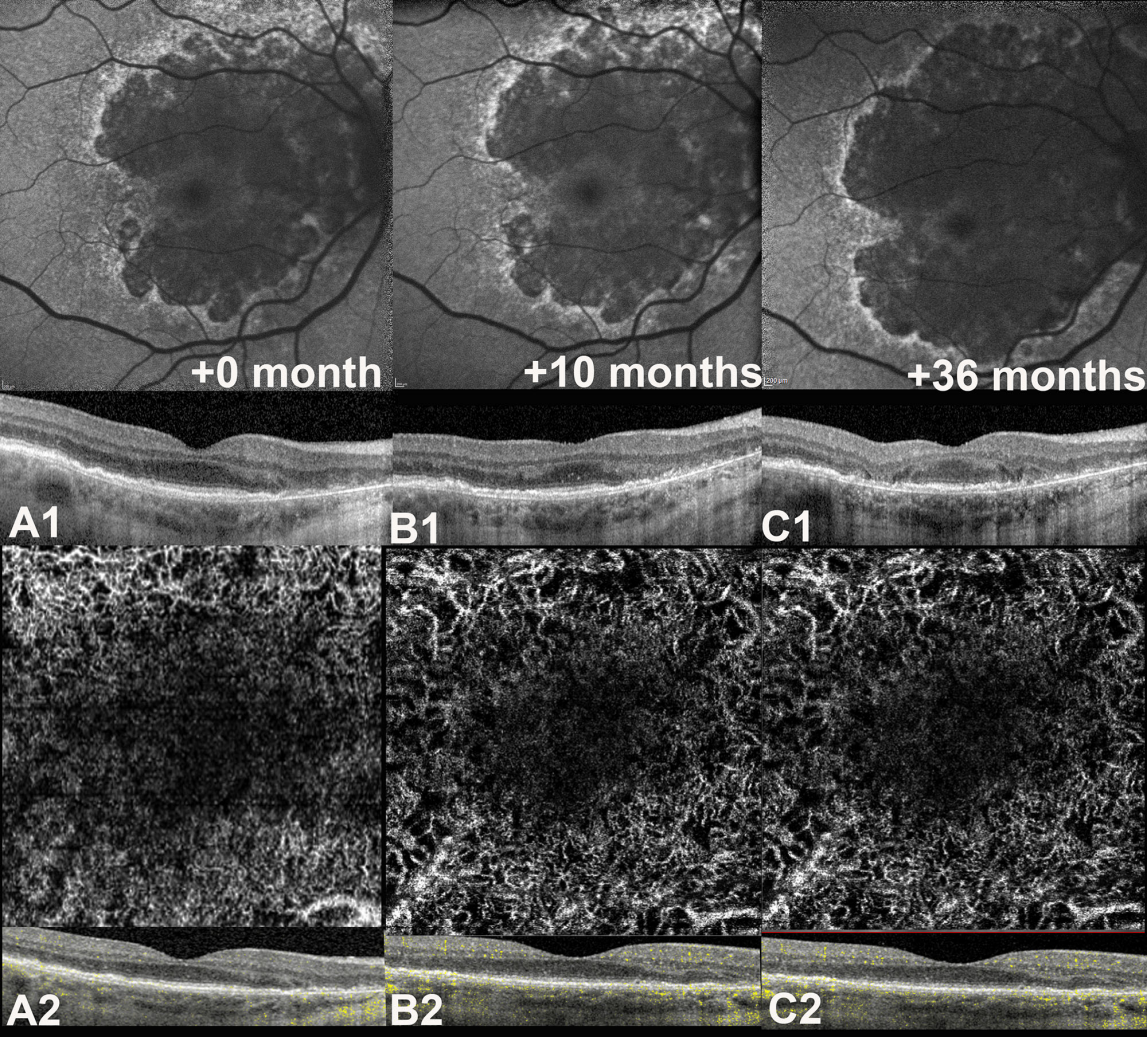
A comparative case with geographic atrophy (GA) with foveal sparing associated with a thin double layer sign (DLS). (**A1**) Fundus autofluorescence (FAF) at baseline shows the atrophic lesions with foveal sparing at baseline confirmed with optical coherence tomography (OCT) B-scan passing through the fovea showing a thin hyporeflective DLS. Noteworthy, despite that the area of foveal sparing seen on OCT B-scan appears similar to that in Figure A1 at baseline, the corresponding estimation of FAF differs substantially, further highlighting the limitations of FAF in delineating GA and foveal sparing in eyes with thick basal laminar deposits (BLamD). (**A2**) OCT angiography (Heidelberg, Germany) of the choriocapillaris slab showing the absence of a neovascular network at the level of thin DLS. (**B1**) FAF at 10 months shows an enlargement of the atrophic lesion with preservation of the foveal sparing area, with a relative stability of the OCT B-scan. (**B2**) OCTA slab is unchanged. (**C1**) At 36 months, the area of atrophy demonstrates further centrifugal enlargement with a marked reduction of the sparing area, as confirmed through OCT B-scan passing through the fovea. A residual hyper-reflective material lying on Bruch's membrane co-localizing with the posterior hypertransmission can be appreciated, a possible signature for persistent BLamD in the atrophic zone. (**C2**) OCTA choriocapillaris slab shows no changes from baseline **A2**.

Repeated-measures ANOVA demonstrated a significant GA area growth for both BlamD (Greenhouse-Geisser corrected, F(1.2, 19.2 = 9.85, *P* = 0.004, ω^2^ = 0.05) and NE-MNV subgroups (F(2, 20) = 27.8, *P* < 0.001, ω^2^ = 0.05) over time. Interestingly, the foveal sparing area demonstrated a significant reduction in the BLamD subgroup (F(1.39, 20.9) = 7.5, *P* < 0.001, ω^2^ = 0.09) but not in the NE-MNV (F(1.2, 11 = 4.15, *P* = 0.06, ω^2^ = 0.02). For further details, refer to exemplary cases in Figures 4 and 5.

## Discussion

The present study proposed to clarify the discrepancies in the prognostic interpretation of DLS in the setting of GA with foveal sparing. Such discrepancies may arise from a non-univocal interpretation of this feature that may represent either thick BLamD or non-exudative type 1 MNV.^11^^,^^17^^,^^20^^,^^31^^–^^33^ To allow more precise discrimination between these two pathologic features, only eyes assessed with OCTA centered on the area of foveal sparing were included. This is particularly important, as the use of OCTA can further probe the nature of DLS, enhancing the accuracy of prediction in GA/cRORA. This has represented an important limitation in previous studies that relied solely on structural OCT.^10^^,^^17^

Our data confirmed that eyes with non-neovascular DLS presented a significantly thinner RPE-BL-BrM splitting (57 vs. 90.4 µm at the highest point on average) compared with eyes with NE-MNV. Furthermore, our results suggest that a threshold of <80 µm may represent a fair trade-off between sensitivity and specificity for identifying thick BLamD. However, the Youden's index of 0.49 indicated only a moderate overall diagnostic effectiveness, as the specificity remained limited, correctly identifying 57.1% of true negative cases (NE-MNV). This further corroborates the importance of a structural OCT-based definition of thin DLS, with OCTA serving as a valuable support in doubtful cases, rather than relying solely on an arbitrary thickness cutoff. The OCT definition of thin DLS, as an in vivo correlate for thick BLamD, is characterized by a single hyporeflective layer that allows a physical distinction between RPE and BrM.^11^^,^^17^^,^^34^ Recently, however, Won et al.^35^ suggested interpreting this signature as a hyporeflective band rather than a split, given that BLamD is continuous and thick in AMD eyes. Another relevant point raised by the authors regards the recognition of these deposits with conventional structural OCT devices, considering that they are usually comprised between 0 and 5 µm. Therefore, it is important to highlight that BLamD can be visualized with commercial OCT devices only when they reach a critical thickness.^34^^,^^36^ On histopathological studies, median BLamD thickness was 4.1 µm in GA eyes,^20^ with an average thickness increasing toward the external limiting membrane (ELM) descent and with RPE dysmorphia (29.2 ± 11.9 µm). In clinical studies, the quantification of thick BLamD measured between splitting borders (RPE-BrM) ranged between 10 and 50 µm with an average comprised between 23.2 and 24.1 µm at the thickest point in rapidly progressing GA phenotypes.^34^^,^^36^ The accumulation of BLamD and membranous debris was hypothesized to represent a survival strategy of dysfunctional RPE. However, the progressive thickening of BLamD further separates the basal RPE surface from the choroidal blood supply, exacerbating RPE ischemia and thus aggravating the RPE degeneration.^37^ Although a minimal RPE functionality is needed to maintain BLamD, when BLamD reaches 20 µm the RPE is already dysfunctional.^20^ Clinically evident BLamD thickening precedes atrophic evolution with perfect topographic correspondence. In vivo observations have shown that as BLamD thickens, the RPE layer exhibits degeneration, characterized by thinning and undulations, which precede RPE loss and increased choroidal hypertransmission.^34^^,^^36^ A thin DLS has been recently identified as one of the most important independent risk factors for atrophy progression from an intermediate AMD stage.^17^^,^^18^^,^^38^

Interestingly, the phenotypic manifestations of thick BLamD in eyes with rapidly progressing atrophy also included the presence of a diffuse trickling pattern on FAF, which appeared to be associated with BLamD accumulation.^34^^,^^36^ The diffuse trickling also exhibited a more grayish appearance, rather than a distinctly black (hypo-autofluorescent) decreased FAF signal.^39^ The leading hypothesis behind this observation is that the presence of thick BLamD may contribute to vitamin A deficiency by impairing the metabolic exchange between the choroid and RPE.^40^ Moreover, BLamD can be intrinsically autofluorescent in the presence of shed granule aggregates within it.^41^^–^^43^ Taken together, these factors may contribute to the grayish FAF signal seen in eyes with thick BLamD, a factor that can make the precise delineation of atrophic areas challenging. In this regard, our results revealed a significant discrepancy between the atrophic areas estimated with FAF and NIR, with an apparent underestimation of the atrophy using FAF. Despite this, the two methods presented an overall acceptable agreement, with an average bias of −0.27. However, this difference could become more relevant in cases involving larger atrophic areas, a consideration that deserves attention when evaluating such patients. Conversely, in our series, no differences were observed between FAF and NIR in assessing foveal sparing. One potential explanation for this is the easier identification of the innermost hypoautofluorescent atrophic border on FAF due to the absence of diffuse trickling, which tends to be more evident at the outer atrophic boundary.

Our data also demonstrate that a thick DLS is likely associated with type 1 MNV, as confirmed through OCTA. A neovascular DLS has been hypothesized to protect the outer retina and RPE by providing metabolic support and oxygenation to the hypoxic RPE, photoreceptors, and outer nuclear layer.^14^^–^^17^ The growth pattern of type 1 MNV suggests a compensatory mechanism aimed at replacing the deficient choriocapillaris, effectively forming a “neochoriocapillaris.”^15^^,^^44^ Indeed, a deficient choroid and choriocapillaris are the main factors involved in the GA expansion.^45^^,^^46^ In this regard, despite a similar GA area at baseline, eyes with type 1 NE-MNV within the area of foveal sparing demonstrated greater preservation of the fovea compared with eyes with BLamD. This finding may further corroborate the potential supportive role of NE-MNV in providing nutritional and metabolic support to the outer retina and RPE, thereby sustaining a central island of functional retina for a long time.^47^ Additionally, it may be conceivable that BLamD may contribute to maintaining type 1 MNV in a non-exudative state by preventing its breakthrough through the RPE into the outer retina.^15^ These hypotheses are further supported by evidence showing no significant change in the foveal sparing area over an average follow-up of 2.3 years in the NE-MNV, compared to a significant reduction in the sparing area observed in the BLamD group after 2.1 years. It is important to highlight, however, that our data indicate similar GA growth rates between the two groups. This finding is plausible, as type 1 MNV was confined to the foveal area, limiting its impact on overall atrophy progression. Noteworthy, 23.8% of eyes with NE-MNV developed exudative changes after a mean of 36.4 ± 24.9 months, highlighting the importance of distinguishing this signature from DLS of non-neovascular origin due to its therapeutic implications.

Although visual acuity (VA) was not an endpoint of the present study, the apparent discrepancy between anatomic foveal preservation in NE-MNV and visual function merits discussion. First, it is important to emphasize that this study was neither designed nor powered to assess functional outcomes. Therefore, factors like unmeasured refractive errors, mild media opacities, or age-related changes could have influenced VA independently of retinal morphology. More importantly, VA alone has been demonstrated to represent an imperfect surrogate for macular function in GA, especially in foveal-sparing and subretinal drusenoid deposit SDD) eyes,^48^^–^^51^ where dissociation between structure and function is well documented. Recent evidence suggests that VA decline occurs before the foveal involvement in GA, suggesting that a significant functional deterioration may precede detectable structural loss.^52^ Furthermore, it has been noted that certain FAF pattern (i.e. diffuse-trickling) can be associated with a more rapid visual deterioration,^52^^,^^53^ potentially reflecting a greater degree of structure-function dissociation in eyes with thick BLamD. Despite this, a near-significant trend toward VA decline was observed in eyes with thick BLamD (*P* = 0.06), opening a window for future prospective studies with appropriate functional testing and larger, stratified cohorts.

Possible limitations of the present study include its retrospective design, which restricted the number of eligible patients, as routine OCTA was not performed for longitudinal GA evaluation in all cases. Furthermore, the resolution of OCTA may be insufficient to detect small neovascular networks, potentially leading to erroneous allocation of the patient into the BLamD group. However, this bias was mitigated by acquiring serial OCTA scans. Another relevant point for future exploration can be the estimation of the GA area in eyes with BLamD, whereas alternative strategies may be needed to avoid inaccurate and false estimations. In this context, the lack of blinding during GA measurements may represent a potential source of bias. This limitation was partially mitigated by using standardized protocols across centers and the resolution of discrepancies through open adjudication by senior retinal specialists. Although the observed baseline difference in the foveal sparing area between the NE-MNV and BLamD groups may be considered a potential confounding factor in the longitudinal analysis, it is also possible that this difference reflects limitations of FAF in estimating GA/sparing in eyes with thick BLamD, particularly because it was not confirmed on NIR imaging or at the 1-year follow-up.

Despite these limitations, the present study presented the main advantage of confirming the presence of a neovascular network within DLS through OCTA. This has permitted a more precise prognostic interpretation of the presence of DLS in eyes with GA. Our results confirm a trend toward relative preservation of the foveal sparing area associated with neovascular tissue, potentially supporting the viability of RPE, photoreceptors, and outer nuclear layer over time. Despite this, it is also important to highlight that this is not offering an advantage in terms of the atrophy growth outside the foveal region, where the neovascular tissue is not providing such metabolic and nutritional support. The neovascular lesion may remain in a non-exudative state thanks to the physical barrier created by BLamD underneath the RPE layer that can avoid the breakthrough of neovessels into it. Our findings may probably offer a better interpretation of the discrepancies in the DLS interpretation observed in previous studies, refining the prognostic role of this signature. Moreover, our results indicate that the presence of BLamD can contribute to an inaccurate estimation of atrophic areas on FAF, likely due to the intrinsic autofluorescence of these sub-RPE deposits. This finding deserves attention in future studies as it may represent a relevant confounder when estimating the atrophic evolution.

In conclusion, the presence of DLS is a relevant predictive factor in the natural history of GA, with a dual interpretation. OCTA proves valuable in distinguishing between BLamD and NE-MNV, but dimensional criteria on structural OCT should be also considered as a helpful distinctive feature. The presence of a type 1 neovascularization offers a potential advantage in these cases by contributing to long-term preservation of the central island of foveal sparing, a region that maintains its anatomic integrity and functionality, positively impacting patients’ daily tasks and quality of life. Although cross-sectional differences in foveal sparing were modest, the longitudinal analysis revealed a significantly greater reduction in the area of foveal sparing over time in the thick BLamD group, suggesting a more aggressive course toward central vision loss in this phenotype. At the same time, the pathogenic distinction of DLS is crucial for the close monitoring of neovascular eyes at risk of developing exudative changes, enabling timely intervention. More importantly, these findings may have future therapeutic implications. The prognostic role of thick BLamD in AMD progression has been largely under-recognized; however, our results suggest it may serve as a critical biomarker and a potential early therapeutic target for slowing disease progression. Future larger, prospective longitudinal studies are encouraged to corroborate these findings and further explore the functional status in eyes with thick BLamD using more sensitive functional tests.
